# Present or Absent: Risks and protective factors of sudden infant death syndrome (SIDS) in the Zambian context

**DOI:** 10.21203/rs.3.rs-6427293/v1

**Published:** 2025-05-13

**Authors:** Ethan M. Zulu, Lawrence Mwananyanda, Rachel C. Pieciak, Leah S. Forman, Janaki Shah, Tim Heeren, Christopher J. Gill, Roma Chilengi, Barbara Payne-Lohman, Cassandra R. Duffy, Godwin Osei-Poku, Donald M. Thea, Somwe Wa Somwe, Julie M. Herlihy

**Affiliations:** Right to Care Zambia; Right to Care Zambia; Boston University School of Public Health; Boston University School of Public Health; Boston University School of Public Health; Boston University School of Public Health; Boston University School of Public Health; Centre for Infectious Disease Research in Zambia; University of Rhode Island; Beth Israel Deaconess Medical Center, Harvard Medical School; Commonwealth of Massachusetts; Boston University School of Public Health; University of Zambia; Boston University School of Public Health

**Keywords:** Sudden infant death syndrome, SIDS, SUID, Infant sleep position, risk factors, breastfeeding, sleep position

## Abstract

**Background:**

Despite a reduction in Sudden Unexplained Infant Death (SUID) in high-income countries, the incidence of SUID and the prevalence of its risk and protective factors remain poorly understood in Zambia due to limited research. The aim of our study was to describe the infant sleep positions and sleep environments in an urban Zambian population to gain a better understanding of the modifiable risk factors for SUID.

**Methods:**

Data from the Zambian Infant Cohort Study (ZICS), a prospective birth cohort, were collected to describe infant sleep practices in Chawama, a densely populated peri-urban community in Lusaka, Zambia. During the 20-week study visit a structured questionnaire was administered to obtain data about the sleeping and environmental risks associated with SUID.

**Results:**

Data were collected from 596 caregivers and 605 infants. Only 6.4% of caregivers did attain an education beyond secondary school, and a significant proportion of infants (20.2%) had low birth weights, with 10.7% of infants confirmed by ultrasound as preterm. Furthermore, 96.5% of infants were placed to sleep on their side or in a prone position, and 98.2% of infants shared a sleep surface with their caregiver. Breastfeeding, a protective factor, was highly prevalent, with 90.2% of infants receiving some form of breastfeeding at the 24-week visit.

**Conclusion:**

The results of this study show that both modifiable (bed-sharing and prone sleep position) and non-modifiable risk factors (low birthweight and prematurity) of SUID are prevalent in this low socioeconomic setting in Zambia. Public health strategies to prevent SUID will need to be innovative and culturally congruent in addressing modifiable risks, such as bedsharing, in settings where there is a lack of space.

**Trial registration:**

Trial number: 1R01HD094650

## BACKGROUND

Sudden infant death syndrome (SIDS) is the sudden death of an infant under 1 year of age without established cause after thorough investigation, including review of clinical history, circumstances of death, and performance of complete autopsy (1). A recent postmortem surveillance study conducted in Zambia found that 7.4% of infant deaths in the community were due to sudden unexplained infant death (SUID), with nearly 5.4% attributed to apparent SIDS (2). This may indicate a significant incidence of suspected SIDS. However, the prevalence of bed-sharing and prone or side sleeping positions, known risk factors for SIDS, is poorly understood in Zambia due to an apparent lack of research interest.

SIDS is a complex phenomenon characterized by multiple interacting risk factors. The triple risk hypothesis suggests that SIDS occurs in infants who have an underlying biological vulnerability and are exposed to an external threat during a critical developmental period. Numerous risk factors for SIDS are well documented globally, including low birth weight, male gender, young maternal age, and multiparity (3). However, research on the prevalence of these non-modifiable risk factors in Zambia is limited to a single cross-sectional survey (4). Additionally, several modifiable factors have been associated with a higher risk of SIDS, such as prone sleeping positions, bedsharing, maternal smoking, and parental alcohol consumption (5). In a recent qualitative study, Osei-Poku et al. (2023) found that mothers viewed the supine sleeping position of their baby as a choking hazard. They also preferred to bedshare with their infants for the convenience of breastfeeding and monitoring the baby (6). Debate persists regarding the potential benefits and risks of shared sleeping practices in relation to SIDS, and there has been little effort to examine the prevalence of these practices in the Zambian context.

Moreover, while a myriad of protective factors, including breastfeeding, pacifier use, and immunization, have been associated with SIDS globally (7), no study in Zambia has comprehensively investigated them, partly due to the sluggish uptake and patchy nature of the investigative process surrounding SIDS in Zambia. This research aims to strengthen Osei-Poku’s earlier cross-sectional study by triangulating data from a prospective birth cohort study and a cross-sectional survey, thereby reducing some of the limitations encountered in his earlier work.

## METHODS

### Population

We investigated infant sleep practices using a structured survey for mothers of 20-week-old infants participating in the Zambian Infant Cohort Study (ZICS). ZICS is a longitudinal observational cohort consisting of 1500 mother-infant dyads in a peri-urban community in Lusaka, Zambia; the methods of ZICS are described elsewhere (8). A structured questionnaire was administered by trained midwives during the 20-week ZICS visit to collect information on the infants’ sleeping environment and position from mothers enrolled in the birth cohort. We obtained written informed consent from every participant. For minors, written informed consent was obtained from their parents or guardians. This study was approved by the Institutional Review Boards of Boston University (H-38119) and University Teaching Hospital (Ref: 007–01-19).

### Analytic method

We provide descriptive statistics (median, interquartile range for continuous variables, and frequencies for categorical variables) regarding the demographic characteristics of mothers and infants, as well as the SIDS risk factors related to the sleeping environment. We report the numbers, percentages, and narratives of verbal autopsies linked to SUID.

## RESULTS

Data were collected from 596 caregivers. The majority of the mothers were between the ages of 25 and 34 years (Table 1a). Most mothers were married, 516/596 (86.6%), and only 6.4% did attain an education beyond secondary school. Alcohol consumption during pregnancy was reported by 12.6% of the women, and smoking was 0.3%. Of the 605 infants included in this study, 50.6% (306/605) were male and 49.4% (299/605) were female (Table 1b). Additionally, 10.7% (65/605) of respondents had preterm infants (< 37 weeks gestation) confirmed by ultrasound, while 20.2% (122/605) of infants were born with low birth weight, and only one was born with a major abnormality. In our cohort, 43.1% of infants were exclusively breastfed for six months, and 90.2% had some breastfeeding at 6 months.

Most infants [98.2% (593/605)] shared a sleep surface with other adults, while a smaller number of babies (1.5%) had room sharing without bedsharing (Table 2). Over three-quarters of babies were placed on their sides to sleep [78.5%, (475/605)], and 18% (109/605) of infants were placed in the prone position. A small proportion of infants were placed in the recommended supine or back sleeping position [3.5%,(21/605)]. When asked about the layers of clothing they use to wrap their babies, nearly three-quarters of infants were bundled in two or more layers [74.2%, (447/605)].

Thirty-six infants died during the study period. After careful adjudication of verbal autopsies and clinical case histories, SUID accounted for 8.3% (3/36) of the deceased infants. A complete verbatim account of the narratives from the two mothers whose babies died of SUID is provided below.

I woke up to check and turn the baby around 01:00 on Monday (15/08/22) but to my surprise the baby was unresponsive, and blood was seen coming out from the nose and mouth with no pulse. We rushed to the hospital here at Chawama and the baby was declared dead around 02:00.

It was shocking. It came so sudden. We didn’t see any signs.

## DISCUSSION

The results of this study, which is the first to triangulate SIDS risk factors from both a birth cohort and a cross-sectional study in Zambia, strengthen the findings that both modifiable and non-modifiable risk factors for SIDS are exceptionally prevalent in low socioeconomic settings. We found a SUID prevalence of 8.3%, along with high rates of bedsharing and lateral/prone sleeping positions, which are significant risk factors for SIDS.

Our results on infant sleeping positions appear consistent with other research that found a high prevalence of lateral and prone positions. These two positions are reported to carry the highest risk of SIDS among infants. Many mothers, however, believe that the prone and lateral positions prevent infants from accidentally aspirating their vomitus during sleep (6). Our cohort also demonstrated a high uptake of breastfeeding. At six months, 43.1% of infants were reported to have exclusive breastfeeding, while 90.2% reported some form of breastfeeding. The extensive health benefits of breastfeeding for both mothers and infants have been widely reported (12). While breastfeeding is protective against SIDS, it also promotes shared sleeping, as evidenced by the high percentage of infants (98.2%) with reported bedsharing. Most researchers studying SIDS agree on the increased risk associated with bedsharing, particularly in conjunction with prematurity and low birth weight (13). However, others hold differing views. For example, proponents of co-sleeping argue that skin-to-skin contact (kangaroo care), especially among preterm and newborn infants, has empirical benefits. They contend that shared sleeping promotes thermoregulation, maternal-infant bonding, and a longer duration of breastfeeding (14). This contradiction complicates health professionals’ ability to offer the best possible advice to mothers.

Room sharing without bedsharing is a recognized practice that significantly reduces SIDS. In contrast, only a very small proportion of mothers reported room sharing in our survey. Our findings support previous observations indicating a low prevalence of room sharing without bed sharing in these disadvantaged communities. Several factors could explain this observation. First, many of these families cannot afford a bed for their baby or a larger house due to their socioeconomic circumstances. Second, the culture of breastfeeding might reinforce bedsharing practices instead of room sharing. Although little is known about how much clothing is required to maintain the infant’s thermal comfort, investigators generally agree that hyperthermia from over bundling should be avoided to reduce the risk of SIDS (15). In our study, nearly three-quarters of mothers reported wrapping their babies in two or more layers of clothing. This finding aligns with Osei-Poku’s (2023) results, which indicated that several participants bundled their babies with at least two blankets during sleep.

Prematurity and low birth weight are well-known risk factors for SIDS. It has been suggested that the immature autonomic system leaves these infants vulnerable to an increased incidence of SIDS. In our study, a substantial percentage of infants (20.2%) were born with low birth weight. This figure is more than double the percentage of low birth weight recorded in Lusaka in the 2022 annual statistical report (11). This difference can be partly attributed to the higher proportion of HIV-infected mothers in our study sample (data not shown here), who are at an increased risk of delivering a low birth weight or premature baby. This, combined with the high rates of bedsharing, puts these infants at an increased risk of SIDS.

It is now well established from a variety of studies that maternal alcohol consumption and smoking increase the risk of SIDS (10). While we found low levels of alcohol consumption and smoking in our cohort of mothers, we cannot draw conclusions about the prevalence of maternal smoking and alcohol consumption among SIDS mothers due to the scarcity of prospectively obtained SIDS incidences. A significant number of mothers in our study did not attain an education level beyond secondary school. This finding was also reported by Osei-Poku et al. (2022). Given that most SIDS prevention campaigns focus on parental education and behavioral modification of infant care, this finding is noteworthy. Educational campaigns for SIDS in Zambia should be customized for mothers with low literacy (9).

### Limitations

It is a limitation of our study that we had to rely on self-reported data, particularly regarding risk factors associated with infant care practices. However, significant strengths of the study include the triangulation of data from the prospective cohort. For instance, we were able to obtain precise birth weight and gestational age by utilizing accurate dating through ultrasound. Furthermore, we gathered more accurate breastfeeding patterns by administering a questionnaire during several routine visits.

## CONCLUSION

This study set out to investigate the risk and protective factors of SIDS in a low socio-economic setting. Our research confirms what others have found recently: that infant sleep and environment-related risk factors for SIDS are highly prevalent in disadvantaged communities. We also noted an increasing number of low birthweight and preterm infants who face a greater risk of SIDS in these vulnerable communities. Future public health promotion will need to address these challenges through carefully designed and targeted education for parents, grandparents, and healthcare providers. What is now needed is a cross-national study involving both rural and urban areas. Indeed, our ongoing study on SIDS (Project Chisoni) already seeks to move beyond observational associations and toward stronger evidence that address issues of categorization and causality.

## Figures and Tables

**Table 1a:** Demographic characteristics of caregivers

Question	Response	Overall

How old are you?	N	596

	Mean (Std Dev)	28 (6)

Categorical age	17-24	172 (28.9%)

	25-34	319 (53.5%)

	35+	105 (17.6%)

2-category marital status	Married	516 (86.6%)

	Not married	79 (13.3%)

	No answer	1 (0.2%)

What was the highest grade you attended?	No formal education	13 (2.2%)

	Primary	145 (24.3%)

	Secondary	400 (67.1%)

	College (and beyond)	38 (6.4%)

Do you smoke cigarettes or use other forms of tobacco?	No	591 (99.3%)

	Yes, including during this pregnancy	2 (0.3%)

	Yes, but stopped in pregnancy	2 (0.3%)

Have you drunk any alcohol since you learned you were pregnant?	No	520 (87.2%)

	Yes	75 (12.6%)

	No answer	1 (0.2%)
	

**Table 1b:** Demographic characteristics of infants

Question	Response	Overall

Total N		605

Sex of baby	Male	306 (50.6%)

	Female	299 (49.4%)

Birthweight (categorical)	> 2500g	478 (79.0%)

	LBW (>1500g - <=2500g	122 (20.2%)

	VLBW (>1000g - <=1500g	5 (0.8%)

Gestational week	Preterm (28 - <34 weeks)	11 (1.8%)

	Late Preterm (34 < 37 weeks)	54 (8.9%)

	Term (37 completed weeks)	540 (89.3%)

Exclusive breastfeeding through 24 weeks	Labor	5 (0.8%)

	Day 6	16 (2.6%)

	Week 6	22 (3.6%)

	Week 10	42 (6.9%)

	Week 14	105 (17.4%)

	Week 20	154 (25.5%)

	Week 24	261 (43.1%)

Any breastfeeding through 6 months	Labor	5 (0.8%)

	Day 6	5 (0.8%)

	Week 6	4 (0.7%)

	Week 10	4 (0.7%)

	Week 14	18 (3.0%)

	Week 20	23 (3.8%)

	Week 24	546 (90.2%)

Major birth anomalies?	No	604 (99.8%)

	Yes	1 (0.2%)
	

**Table 2:** Risk factors for SIDS

Question	Response	Overall

Total N		605

Child age in days at post-natal visit	N	605

	Mean (Std Dev)	148 (20)

Over the last two weeks, in what position have you USUALLY placed your baby to sleep?	On the infant’s side (lateral)	475 (78.5%)

	On the infant’s stomach (prone)	109 (18.0%)

	On the infant’s back (supine)	21 (3.5%)

Where does baby USUALLY sleep?	In a parent’s (or other adult’s) room on his/her own mattress or pad	9 (1.5%)

	In a parent’s (or other adult’s) bed with adult(s)	593 (98.2%)

	In another child’s room on his/her own mattress or pad	2 (0.3%)

Over the last two weeks, what type of outfit did you USUALLY put your baby in for sleeping? Note: Key is to assess how many layers of clothes or bundling is occurring	Just a nappy	54 (9.0%)

	One light layer of clothes	102 (16.9%)

	Two layers of clothes (for example with romper)	279 (46.3%)

	Two layers of clothes plus baby tightly swaddled in chitenge	53 (8.8%)

	Multiple layers including a blanket or shawl	115 (19.1%)
	

## Data Availability

Data that support the results of this study is available on BEDAC repository and can be shared upon request.

## Abbreviations

SIDS: Sudden Infant Death Syndrome
SUID: Sudden Unexpected Infant Death
MoH: Ministry of Health
HIV: Human Immune Virus
